# Study on the Thermohydrodynamic Friction Characteristics of Surface-Textured Valve Plate of Axial Piston Pumps

**DOI:** 10.3390/mi13111891

**Published:** 2022-11-02

**Authors:** Zhaoqiang Wang, Lingtao Sun, Bo Han, Xiaoqiang Wang, Zhiwei Ge

**Affiliations:** 1 School of Mechanical and Automotive Engineering, Shanghai University of Engineering Science, Shanghai 201620, China; 2 Shanghai Marine Equipment Research Institute, Shanghai 200031, China; 3 Shanghai Hydronew Hydraulic Mechanical and Electronic Engineering Co., Ltd., Shanghai 200071, China; 4 Shanghai Electric Hydraulics & Pneumatics Co., Ltd., Shanghai 200237, China

**Keywords:** axial piston pump, micro-textured valve plate, thermohydrodynamic, oil film, friction characteristics

## Abstract

The purpose of this paper is to study the oil film and friction characteristics of valve plates with a micro-textured surface and to explore the influence of textures of different shapes and sizes on the valve plates. Firstly, on the basis of thermohydrodynamic theory, this paper established the lubrication model of the oil film on the valve plate pair of swashplate axial piston pumps, according to the Reynolds equation. Secondly, the micro-texture was added to the mathematical model of the valve plate pair’s oil film. A combination of the energy equation, oil-film-thickness equation, elastic deformation equation, viscosity–pressure and viscosity–temperature equation, the finite difference method, as well as the relaxation iteration method, was used to solve the problem, and the textured and non-textured valve plate surfaces were simulated. The nephogram of the oil-film-thickness distribution, elastic deformation distribution, oil-film-pressure distribution and oil-film-temperature distribution were generated. Then, the control variable method was used to change the cylinder rotational speed, tilt angle, oil viscosity, initial oil film thickness and other parameters to analyze their effects on oil film characteristics. In addition, the friction characteristics of non-textured surfaces, square textured surfaces, triangular textured surfaces and circular textured surfaces were compared and analyzed. It was found that the textured surface of valve plates can obviously improve friction efficiency under the same operating conditions. The square texture, especially, is the preferable shape, rather than the triangular texture and the circular texture, and the friction performance is at its best when the texture depths are between 20 μm and 50 μm. The results provide a theoretical basis for the design and improvement of the valve plate.

## 1. Introduction

Axial piston pumps are the key components in many hydraulic systems, which are widely used in harsh operating conditions because they can run efficiently under high pressure and at various speeds [1,2]. However, during the operation of axial piston pumps, the power loss of the valve plate can reach up to 8%. The main sources of power loss are the friction and leakage of the lubrication interface: the former can reach 5% and the latter can reach 3% [3,4]. The friction loss is not only related to the viscosity of hydraulic oil, but also to the textured surface of the lubrication interface [5]. Understanding how to reduce power loss caused by the valve plate and the design of the lubrication interface are key to improving the efficiency of the piston pump. The surface-texturing technique is a new technology in the field of tribology [6]. It has obvious positive effects in improving the load-carrying capacity, wear resistance and friction efficiency of friction pairs, and this is widely recognized.

Tribology researchers all over the world have carried out extensive research on the processing, application, lubrication mechanism and optimization design of micro-pit surface textures, and they have obtained a series of achievements in recent decades. It has been found that micro-textured surfaces, such as grooves [7], asperities [8], and dimples [9], can also improve lubrication performance and reduce friction loss. In 2007, French scholar Tala-Ighil studied the radial bearing with a spherical textured surface and found that the friction performance is related to the area ratio, depths and diameter of the micro-texture [10]. From 2009 to 2013, Wang and Yu conducted a series of experiments and concluded that there is an optimal texture radius that makes the texture effect most obvious compared to the experimental data. In particular, to textures with different shapes, there is an optimal micro-pit area ratio and depth, corresponding to the largest effect [11,12,13,14]. In 2011, American experts found that textured surfaces can always reduce the friction coefficient to varying degrees [15]. In 2013, Muthuvel and Rajagopal, as well as Scaraggi and Segu, explored the relationship between micro-textures with different parameters and friction properties and proved that micro-textures can effectively reduce friction coefficients and improve wear resistance [16,17,18,19]. From Jiangsu University, Fu established a mathematical model of hydrodynamic lubrication for parabolic, triangular and rectangular micro-textured surfaces along a uniform straight line [20,21]. The equations of oil film thickness and pressure were derived, then the boundary conditions were given, and the pressure distribution was solved by using the multigrid method. In 2018, Zhang used laser surface texturing (LST) to process micro-textures on a brass column and carried out experimental research on an EHA pump prototype [22]. It was proved that the texture can improve mechanical efficiency and prototype efficiency, and it was explained that the texture can improve the angle by reducing wear and cylinder inclination. The next year, Chen optimized the micro-texture, further improved the efficiency of the pump, and evaluated the parameters of the texture [23]. In 2020, Ye found a textured slipper with an area density and a dimple-depth-to-diameter ratio of 24% and 0.3, which can provide a greater load-carrying capacity when taking into account the textured-surface deformation [24].

In recent years, there has been a lot of research on the lubrication models of the valve plate pair of pumps. Bergada pointed out the relationship between oil-film-thickness and oil-film-pressure distribution and studied the leakage between the cylinder block and the valve plate [25,26]. According to the Reynolds equation, Chao derived the pressure formula of the valve plate pair and the distribution of the pressure and temperature was analyzed numerically [27]. Lin used computational fluid dynamics (CFD) software to analyze the pressure and temperature of the oil film [28]. In summary, the effect of micro-texture on friction efficiency was studied. However, there are few studies on the optimization of texture structure.

In this paper, the authors established a coupled numerical lubrication model of a cylinder block and the valve plate of an axial piston pump, based on considerations of rectangle micro-textures. Combined with thermohydrodynamic theory, the finite difference method was used to solve the Reynolds equation and energy equation by using the difference quotient of pressure and temperature between nodes, instead of reciprocals. Then, the discrete pressure and temperature values were calculated by FORTRAN (FORTRAN95, IBM, Armonk, NY, USA) procedures. Through continuous iterative coupling, the accuracy of the calculation results was improved and the numerical results within the allowable error range was obtained. Additionally, the nephograms of pressure, temperature and elastic deformation distribution were made by MATLAB (MATLAB2018, MathWorks, Natick, MA, USA). This makes it convenient for us to observe the change trend.

Finally, by means of the control variable method, the influence of the cylinder rotational speed, tilt angle, oil viscosity, thickness, texture lengths and depths on the friction characteristics of non-textured and textured surfaces of the valve plate were calculated and analyzed. These conclusions lay a foundation for improving the design of valve plates.

## 2. Model and Theory 

### 2.1. Model Building

**Figure 1:**
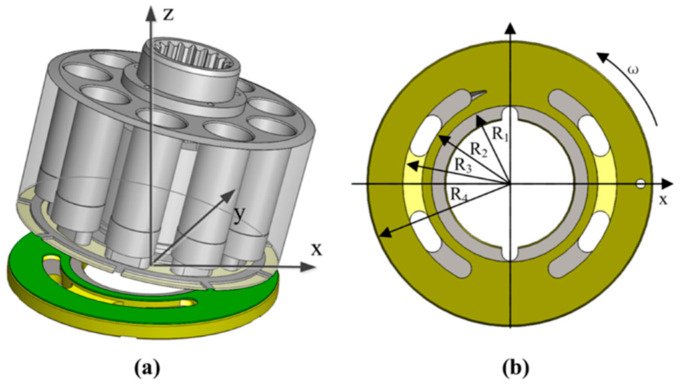
Model of valve plate pair of the piston pump. (**a**) Structure diagram; (**b**) Schematic of the main dimensions.

During the operation of the piston pump, due to the high-speed rotation of the cylinder block relative to the valve plate, the offset load moment on the cylinder block makes it tilt to one side along a certain central axis, as shown in Figure 1a. The friction interface between the cylinder block and the valve plate is annular, and there are regularly arranged micro-pits on the friction surface of the valve plate, the size of which is shown in Figure 1b. In order to calculate the annular oil film characteristics conveniently, the rectangular coordinate system is transformed into a polar coordinate system, and the conversion rules are as follows:

(1) $$\{\begin{array}{l} x=r\theta ,\;dx=rd\theta \\ y=r,dy=dr\\ z=z \end{array}$$

θ = circumference angle

r = radius

Assuming that the inclination angle between the cylinder block and the valve plate is φ, the following oil-film-thickness formula can be obtained:

(2) $$h=h_{0}+r\cdot sin\theta \cdot tan\varphi$$

h = thickness of oil film

$h_{0}$ = initial oil film thickness

φ = cylinder block tilt angle

**Figure 2:**
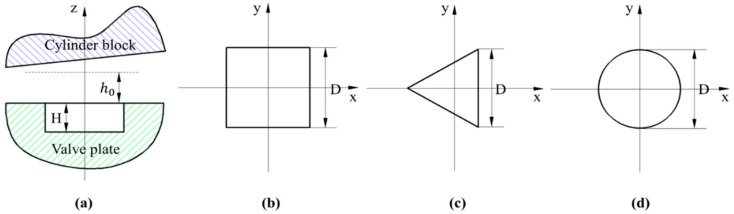
Textures of three different shapes. (**a**) The size of texture; (**b**) Square texture; (**c**) Triangular texture; (**d**) Circular texture.

The micro-pits are arranged regularly on the friction surface of the valve plate. The shape and size of the texture are shown in Figure 2. It can be seen from Figure 2a that the depth of the texture is H. Figure 2b shows a square texture, and its side length is D; Figure 2c shows a triangular texture, and its side length is D; and Figure 2d shows a circular texture and its radius is D.

The Reynolds equation in this study is derived from the Navier–Stokes equation for incompressible flow; therefore, it is necessary to make assumptions about the oil film in the valve plate pair before solving the Reynolds equation. The assumptions are as follows:

- Lubricating oil is treated as an incompressible fluid.
- The lubricating oil is Newtonian fluid.
- The inertial force can be ignored.
- Oil film pressure remains constant along the z-axis.

### 2.2. Solving Formula of Theory

The oil film pressure of the valve plate pair is governed by the Reynolds equation and the generalized Reynolds equation is expressed as [29]:

(3) $$\frac{\partial }{\partial x}\left(\frac{\rho h^{3}}{\eta }\frac{\partial p}{\partial x}\right)+\frac{\partial }{\partial y}\left(\frac{\rho h^{3}}{\eta }\frac{\partial p}{\partial y}\right)=6\left[\frac{\partial }{\partial x}\left(\rho hU\right)+\frac{\partial }{\partial y}\left(\rho hV\right)+2\rho \left(w_{h}-w_{0}\right)\right]$$

According to the assumptions in Section 2.1, Equation (3) can be simplified, obtaining a simplified Reynolds equation as:

(4) $$\frac{\partial }{\partial x}\left(\frac{h^{3}}{\eta }\frac{\partial p}{\partial x}\right)+\frac{\partial }{\partial y}\left(\frac{h^{3}}{\eta }\frac{\partial p}{\partial y}\right)=6U\frac{\partial h}{\partial x}$$

The simplified Reynolds equation in rectangular coordinates is transformed into a form of polar coordinates. Equation (1) can be replaced with Equation (4) to obtain the following formula:

(5) $$\frac{1}{r}\frac{\partial }{\partial \theta }\left(\frac{h^{3}}{\eta }\frac{\partial p}{\partial \theta }\right)+\frac{\partial }{\partial r}\left(\frac{rh^{3}}{\eta }\frac{\partial p}{\partial r}\right)=6\omega r\frac{\partial h}{\partial \theta }$$

p = working pressure

η = viscosity of oil film

ω = velocity of rotation

Discretize Equation (5):

(6) $$\frac{h_{i+0.5,j}^{3}}{r_{i,j}\eta \Delta \theta^{2}}p_{i+1,j}-\frac{h_{i+0.5,j}^{3}}{r_{i,j}\eta \Delta \theta^{2}}p_{i,j}-\frac{h_{i-0.5,j}^{3}}{r_{i,j}\eta \Delta \theta^{2}}p_{i,j}+\frac{h_{i-0.5,j}^{3}}{r_{i,j}\eta \Delta \theta^{2}}p_{i-1,j}+\frac{r_{i,j+0.5}h_{i,j+0.5}^{3}}{\eta \Delta r^{2}}p_{i,j+1}\;\;\;\;\;-\frac{r_{i,j+0.5}h_{i,j+0.5}^{3}}{\eta \Delta r^{2}}p_{i,j}-\frac{r_{i,j-0.5}h_{i,j-0.5}^{3}}{\eta \Delta r^{2}}p_{i,j}+\frac{r_{i,j-0.5}h_{i,j-0.5}^{3}}{\eta \Delta r^{2}}p_{i,j-1}\;=6\omega r_{i,j}\frac{h_{i+0.5,j}-h_{i-0.5,j}}{\Delta \theta }\;\;\;\;\;\;\;\;\;\;\;\;$$

Presume that:

(7) $$\left\{ \begin{array}{l} A_{1}=\frac{r_{i,j+0.5}h_{i,j+0.5}^{3}}{\eta \Delta r^{2}}\\ B_{1}=\frac{r_{i,j-0.5}h_{i,j-0.5}^{3}}{\eta \Delta r^{2}}\\ C_{1}=\frac{h_{i+0.5,j}^{3}}{r_{i,j}\eta \Delta \theta^{2}}\\ D_{1}=\frac{h_{i-0.5,j}^{3}}{r_{i,j}\eta \Delta \theta^{2}}\\ E_{1}=A_{1}+B_{1}+C_{1}+D_{1}\\ F_{1}=6\omega r_{i,j}\frac{h_{i+0.5,j}-h_{i-0.5,j}}{\Delta \theta } \end{array}\right.$$

Equation (5) can be written as follows:

(8) $$A_{1}p_{i,j+1}+B_{1}p_{i,j-1}+C_{1}p_{i+1,j}+D_{1}p_{i-1,j}-\left(A_{1}+B_{1}+C_{1}+D_{1}\right)p_{i,j}=F_{1}$$

(9) $$p_{i,j}=\frac{A_{1}p_{i,j+1}+B_{1}p_{i,j-1}+C_{1}p_{i+1,j}+D_{1}p_{i-1,j}-F_{1}}{E_{1}}$$

The temperature distribution of the lubricating oil film can be obtained by numerically solving the energy equation, and the two-dimensional energy equation can be taken as:

(10) $$Q_{x}\frac{\partial T}{\partial x}+Q_{y}\frac{\partial T}{\partial y}=\frac{h^{3}}{12\eta J\rho C_{\rho }}\left[{\left(\frac{\partial p}{\partial x}\right)}^{2}+{\left(\frac{\partial p}{\partial y}\right)}^{2}\right]+\frac{\eta U^{2}}{hJ\rho C_{\rho }}$$

Trans Equation (10), which changed rectangular coordinates to polar coordinates, obtains this:

(11) $$\frac{\partial T}{\partial \theta }=\frac{r}{Q_{x}}\left(-Q_{y}\frac{\partial T}{\partial r}+\frac{\frac{\eta }{h}{\left(\omega r\right)}^{2}+\frac{h^{3}}{12\eta }\left[{\left(\frac{\partial p}{r\partial \theta }\right)}^{2}+{\left(\frac{\partial p}{\partial r}\right)}^{2}\right]}{J\rho C_{\rho }}\right)$$

T = working temperature of oil

$Q_{x}\;$= volume flowrate in X direction

$Q_{y}\;$= volume flowrate in Y direction

J = mechanical equivalent of heat

ρ = density of lubricating oil

$C_{p}\;$= specific heat capacity of lubricating oil

Discretize Equation (11):

(12) $$T_{i,j}=T_{i-1,j}+\frac{-r_{i,j}{\left(Q_{y}\right)}_{i,j}\Delta \theta }{{\left(Q_{x}\right)}_{i,j}\Delta r}\left(T_{i,j}-T_{i,j-1}\right)\;\;\;\;\;\;\;\;\;\;\;\;\;\;\;\;\;\;\;+\frac{r_{i,j}\Delta \theta }{{\left(Q_{x}\right)}_{i,j}}\;\left(\frac{\frac{\eta_{i,j}}{h_{i,j}}{\left(\omega r_{i,j}\right)}^{2}+\frac{h_{i,j}^{3}}{12\eta_{i,j}}\left[{\left(\frac{\partial p}{r_{i,j}\partial \theta }\right)}^{2}+{\left(\frac{\partial p}{\partial r}\right)}^{2}\right]}{J\rho C_{\rho }}\right)$$

Presume that:

(13) $$\left\{ \begin{array}{l} A_{2}=J\rho C_{\rho }\;\\ B_{2}=\frac{\eta_{i,j}}{h_{i,j}}{\left(\omega r_{i,j}\right)}^{2}+\frac{h_{i,j}^{3}}{12\eta_{i,j}}\left[{\left(\frac{\partial p}{r_{i,j}\partial \theta }\right)}^{2}+{\left(\frac{\partial p}{\partial r}\right)}^{2}\right]\\ C_{2}=\frac{-r_{i,j}{\left(Q_{y}\right)}_{i,j}\Delta \theta }{{\left(Q_{x}\right)}_{i,j}\Delta r}\\ D_{2}=\frac{r_{i,j}\Delta \theta }{{\left(Q_{x}\right)}_{i,j}}\\ E_{2}=T_{i-1,j}-C_{2}\times T_{i,j-1}\\ {\left(Q_{x}\right)}_{i,j}=\frac{h_{i,j}\omega r_{i,j}}{2}-\frac{h_{i,j}^{3}}{12\eta_{i,j}}\frac{p_{i,j}-p_{i-1,j}}{r_{i,j}\Delta \theta }\\ {\left(Q_{y}\right)}_{i,j}=-\frac{h_{i,j}^{3}}{12\eta_{i,j}}\frac{p_{i,j}-p_{i,j-1}}{\Delta r} \end{array}\right.$$

Equation (12) can be simplified as:

(14) $$T_{i,j}=\frac{\left(E_{2}+\frac{D_{2}B_{2}}{A_{2}}\right)}{1-C_{2}}$$

Boundary conditions:

- The initial temperature of the oil film is equal to the ambient temperature.
- The boundary of the oil film is insulation.

Under the influence of pressure and temperature, the viscosity of the lubricating oil will also change. It is usually expressed by Roelands formula:

(15) $$\eta =\eta_{0}exp\left\{\left(ln\eta_{0}+9.67\right)\left[{\left(1+5.1\times 10^{-9}p\right)}^{0.68}+{\left(\frac{T-138}{T_{0}-138}\right)}^{-1.1}-1\right]\right\}$$

η = viscosity of oil film

$\eta_{0}$ = initial viscosity of oil film

$T_{0}$ = initial temperature of oil

**Figure 3:**
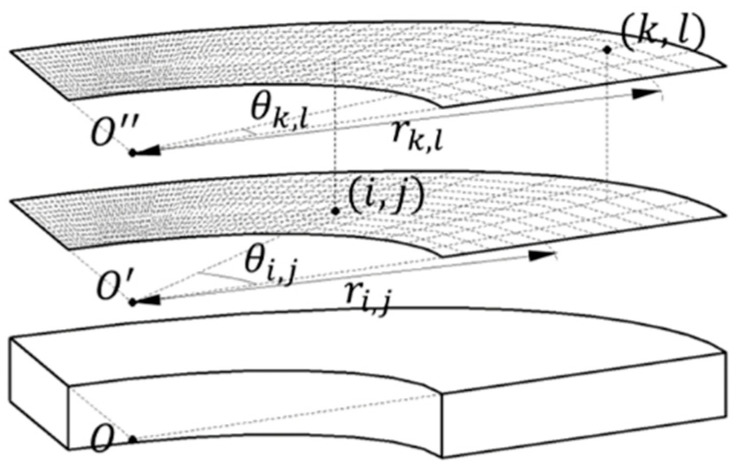
Valve plate under the influence of pressure.

The wedge of the oil film is formed by the inclination of the cylinder block that provides the base for the hydrodynamic effect. As a result, the distribution of oil film pressure is single-peak-like, concentrated on one side of the oil film. Due to the concentration of pressure distribution, the valve plate has an elastic deformation, which needs to be calculated according to the elastic deformation theory. As shown in Figure 3, the elastic deformation $\delta_{ij}^{kl}$ of node (i,j) in plane $X^{\prime }O^{\prime }Y^{\prime }$ caused by node (k,l) in plane $X^{\prime \prime }O^{\prime \prime }Y^{\prime \prime }$ was taken as:

(16) $$\delta_{ij}^{kl}=\frac{2}{\pi S}\iint \frac{p_{k,l}}{\sqrt{{\left(X_{i}^{\prime }-X_{k}^{\prime \prime }\right)}^{2}+{\left(Y_{j}^{\prime }-Y_{l}^{\prime \prime }\right)}^{2}}}dxdy$$

Convert the coordinate system to a cylindrical coordinate system and discretize Equation (16):

(17) $$\delta_{ij}^{kl}=\frac{2}{\pi S}\frac{p_{k,l}r_{k,l}\Delta \theta \Delta r}{\sqrt{{\left[\frac{r_{i,j}\left|\theta_{i,j}-\theta_{k,l}\right|+r_{k,l}\left|\theta_{i,j}-\theta_{k,l}\right|}{2}\right]}^{2}+{\left(r_{i,j}-r_{k,l}\right)}^{2}}}$$

When nodes (i,j) and (k,l) are the same point, replace node (i,j−1) with (k,l) in the process of calculating the distance:

(18) $$\delta_{ij}^{kl}\approx \frac{2}{\pi S}\iint \frac{p_{i,j}r_{i,j}\Delta \theta \Delta r}{\sqrt{{\left[\frac{r_{i,j}\left|\theta_{i,j}-\theta_{i,j-1}\right|+r_{i,j-1}\left|\theta_{i,j}-\theta_{i,j-1}\right|}{2}\right]}^{2}+{\left(r_{i,j}-r_{i,j-1}\right)}^{2}}}=\frac{2}{\pi S}\frac{p_{i,j}r_{i,j}\Delta \theta \Delta r}{\Delta r}$$

Therefore, the total elastic deformation at node (i,j) is as follows:

(19) $$\delta_{i,j}=\frac{2}{\pi S}\frac{p_{i,j}r_{i,j}\Delta \theta \Delta r}{\Delta r}+\frac{2}{\pi S}\underset{k=1}{\sum^{N}}\underset{l=1}{\sum^{M}}\frac{p_{k,l}r_{k,l}\Delta \theta \Delta r}{\sqrt{{\left[\frac{r_{i,j}\left|\theta_{i,j}-\theta_{k,l}\right|+r_{k,l}\left|\theta_{i,j}-\theta_{k,l}\right|}{2}\right]}^{2}+{\left(r_{i,j}-r_{k,l}\right)}^{2}}}$$

where $\frac{1}{S}=\frac{1}{2}\left(\frac{1-V_{2}{}^{2}}{S_{2}}+\frac{1-V_{1}{}^{2}}{S_{1}}\right)$

δ = elastic deformation

N = number of nodes in circumferential direction

M = number of nodes in radius direction

S = comprehensive elastic modulus

$S_{1}$ = elastic modulus of the valve plate

$S_{2}$ = elastic modulus of the cylinder block

$V_{1}$ = Poisson’s ratio of valve plate

$V_{2}$ = Poisson’s ratio of cylinder block

Presume that:

(20) $$\{\begin{array}{l} A_{3}=\frac{2}{\pi S}=\frac{1}{\pi }\left(\frac{1-V_{2}{}^{2}}{S_{2}}+\frac{1-V_{1}{}^{2}}{S_{1}}\right)\\ B_{3}=\frac{r_{k,l}\Delta \theta \Delta r}{\sqrt{{\left[\frac{r_{i,j}\left|\theta_{i,j}-\theta_{k,l}\right|+r_{k,l}\left|\theta_{i,j}-\theta_{k,l}\right|}{2}\right]}^{2}+{\left(r_{i,j}-r_{k,l}\right)}^{2}}}\\ C_{3}=r_{i,j}\Delta \theta \end{array}$$

Simplify Equation (18) as follows:

(21) $$\delta_{i,j}=A_{3}C_{3}p_{i,j}+A_{3}\underset{k=1}{\sum^{N}}\underset{l=1}{\sum^{M}}B_{3}\cdot p_{k,l}$$

### 2.3. Numerical Calculations

In this paper, the finite difference method is used to solve the mathematical model. The Reynolds equation and energy equation are discretized by the central difference method. More concretely, difference coefficients are used to replace derivatives of the Reynolds equation and energy equation, which improves the accuracy of the calculation. Concurrently, the numerical solutions of pressure and temperature of oil film are obtained by the relaxation iteration method. The convergence condition is taken as:

(22) $$\frac{\sum_{i=1}^{N}\sum_{j=1}^{M}\left(p_{i,j}^{k+1}-p_{i,j}^{k}\right)}{\sum_{i=1}^{N}\sum_{j=1}^{M}p_{i,j}^{k+1}}\leq Err$$

where Err is an accuracy error of $10^{-5}$ and k is the number of iterations.

Firstly, the initial conditions of the valve plate parameters and the operating conditions are set. Secondly, according to the oil-film-thickness formula, the oil film thickness of each node is calculated, which is substituted into the Reynolds equation to calculate the pressure distribution of the lubricating oil film. According to the pressure of each node, the elastic deformation of the valve plate is calculated by substituting it into the elastic deformation equation. Finally, the obtained numerical solution is brought into the energy equation to obtain the temperature distribution of the lubricating oil film. At this time, it is judged whether the numerical solution meets the required accuracy requirements. If it does not meet the requirements, the results will be brought back into the calculation.

**Figure 4:**
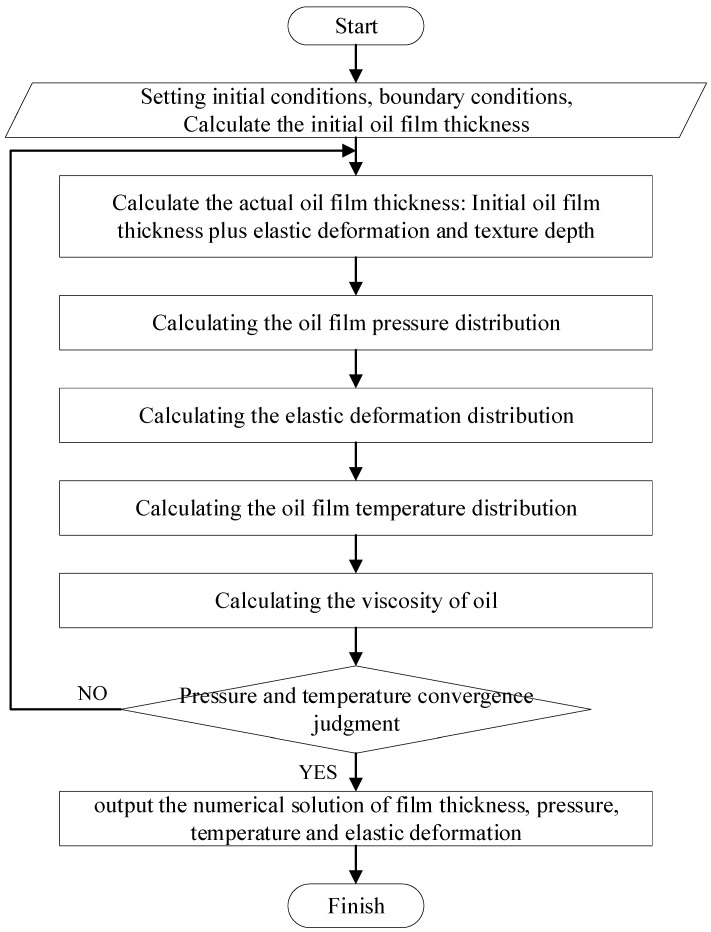
Flowchart of the numerical calculation procedures.

The flowchart of the numerical calculation procedures for oil film pressure, temperature and elastic deformation is shown in Figure 4.

## 3. Oil Film Characteristics

In order to obtain the difference between the lubrication characteristics of the valve plate surface that are textured and non-textured, the square texture is taken as an example for comparative analysis. The square textured surface of the valve plate with a 90×6 regular distribution was selected as the control group, 90 micro-pits were distributed in the circumferential direction and 6 micro-pits were distributed in the radius direction. The specific initial parameters are shown in Table 1:

### 3.1. Nephogram of Oil-Film-Thickness Distribution 

**Figure 5:**
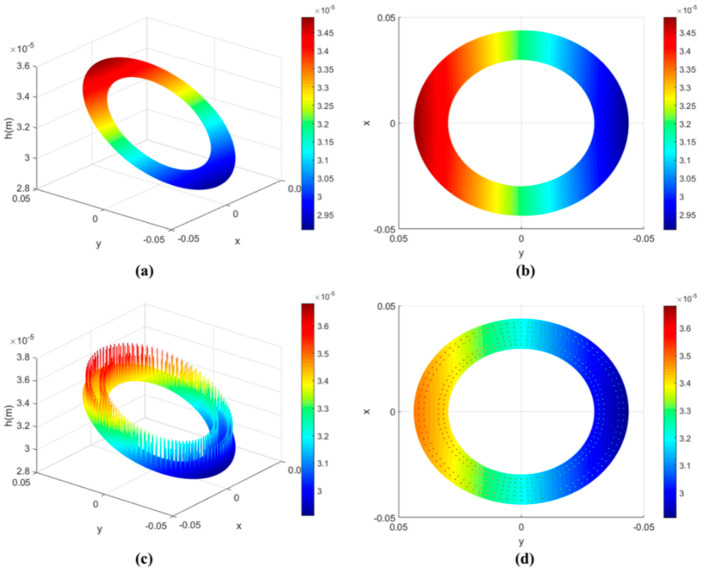
Oil-film-thickness distribution. (**a**) 3D diagram of the distribution of oil film thickness on the non-textured surface of the valve plate; (**b**) Top view of the distribution of oil film thickness on the non-textured surface of the valve plate; (**c**) 3D diagram of the distribution of oil film thickness on the square; (**d**) Top view of the distribution of oil film thickness on the square textured surface of the valve plate.

The wedge-shaped oil film is thicker on one side and thinner on the other, affected by the inclined cylinder block, which can be found in Figure 5. The result is basically consistent with the previous analysis of the mathematical model. 

Figure 5a,b show the distribution of oil film thickness on the non-textured surface of the valve plate. It can be seen from the figure that, due to the inclination of the cylinder block, the oil-film-thickness distribution is uneven, and its thickness changes with the change in circumferential angle. Figure 5c,d show the distribution of oil film thickness on the square textured surface of the valve plate. Compared with the nephogram of oil-film-thickness distribution on the non-textured surface, it is found that the overall distribution of oil film thickness is similar, but the oil film thickness in the textured region increases the depth of the texture.

### 3.2. Nephogram of Oil-Film-Pressure Distribution

**Figure 6:**
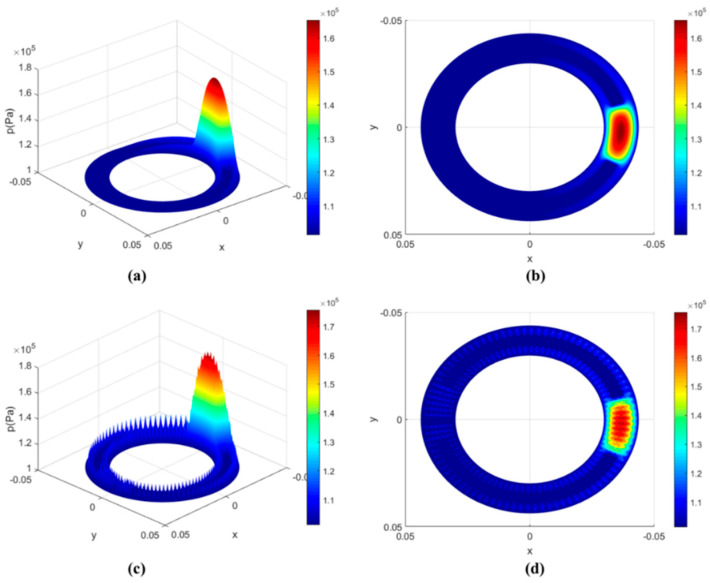
Oil-film-pressure distribution. (**a**) 3D diagram of the distribution of oil film pressure on the non-textured surface of the valve plate; (**b**) Top view of the distribution of oil film pressure on the non-textured surface of the valve plate; (**c**) 3D diagram of the distribution of oil film pressure on the square textured surface of the valve plate; (**d**) Top view of the distribution of oil film pressure on the square textured surface of the valve plate.

Because of the rotation of the cylinder block relative to the valve plate and the formation of the wedge-shaped oil film, the foundation for the hydrodynamic effect is provided, and the hydrodynamic effect mainly occurs in the convergence zone of the oil film gap. Figure 6a,b show the distribution of oil film pressure on the non-textured surface of the valve plate. Figure 6c,d show the distribution of oil film pressure on the square textured surface of the valve plate. Compared with the oil-film-pressure distribution nephogram of the non-textured surface port pair, there are obvious pressure peaks in the micro-textured area, and the oil film pressure is higher than that in the surrounding non-textured area. These oil film pressures are transformed into pushing forces acting on the cylinder block, pushing the cylinder block away from the port plate, reducing the contact between the cylinder block and the port plate and reducing the wear in the friction process, improving its friction efficiency.

### 3.3. Nephogram of Elastic Deformation Distribution

**Figure 7:**
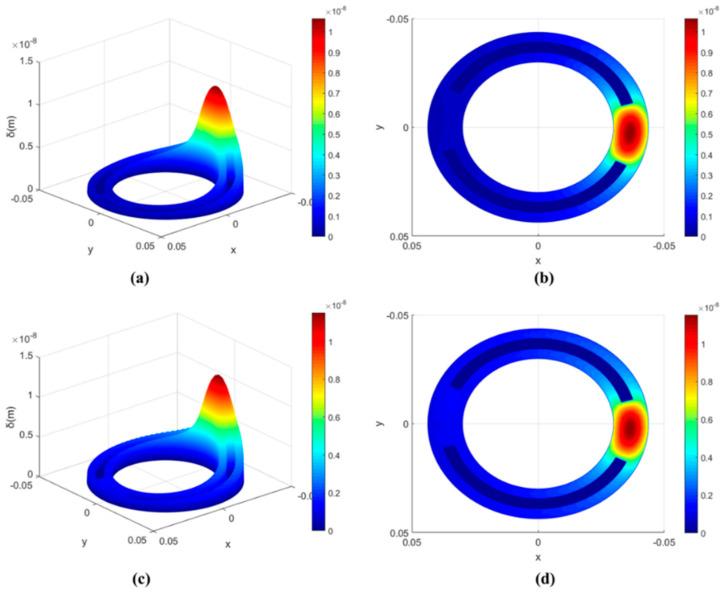
Elastic deformation distribution. (**a**) 3D diagram of the elastic deformation distribution on the non-textured surface of the valve plate; (**b**) Top view of the elastic deformation distribution on the non-textured surface of the valve plate; (**c**) 3D diagram of the elastic deformation distribution on the square textured surface of the valve plate; (**d**) Top view of the elastic deformation distribution on the square textured surface of the valve plate.

Figure 7 shows the elastic deformation distribution of the valve plate. Elastic deformation is mainly caused by the oil film pressure, so the distribution of the elastic deformation is concentrated on the side with a smaller oil film thickness as the oil film pressure.

Figure 7a,b shows the distribution of elastic deformation on the non-textured surface of the valve plate. Figure 7c,d show the distribution of elastic deformation on the square textured surface of the valve plate.

### 3.4. Nephogram of Oil-Film-Temperature Distribution

**Figure 8:**
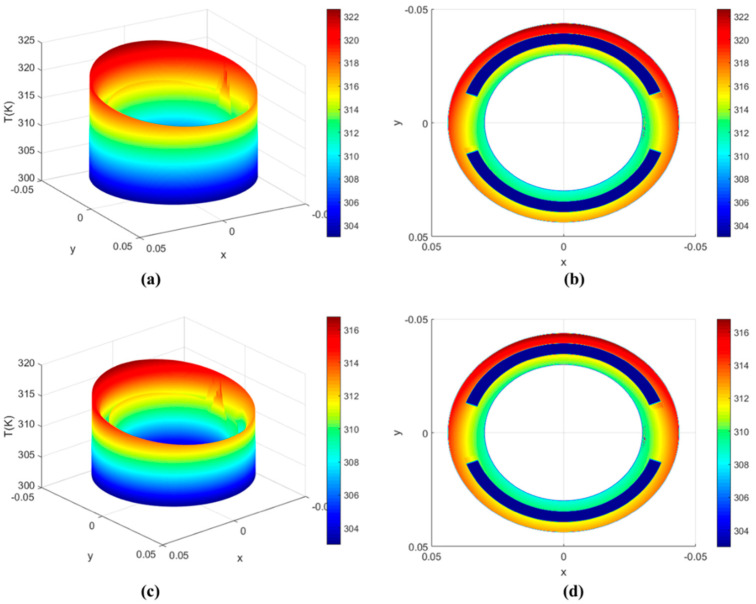
Oil-film-temperature distribution. (**a**) 3D diagram of the distribution of temperature on the non-textured surface of the valve plate; (**b**) Top view of the distribution of temperature on the non-textured surface of the valve plate; (**c**) 3D diagram of the distribution of temperature on the square textured surface of the valve plate; (**d**) Top view of the distribution of temperature on the square textured surface of the valve plate.

Figure 8 shows the oil-film-temperature distribution. Figure 8a,b show the distribution of temperature on the non-textured surface of the valve plate. Figure 8c,d show the distribution of temperature on the square textured surface of the valve plate.

**Figure 9:**
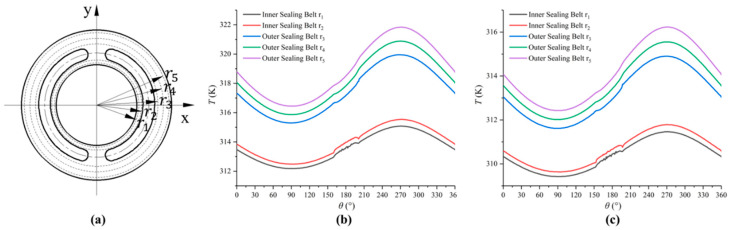
Oil-film-temperature distribution in different radii. (**a**) The positions of five circles with different radii; (**b**) The temperature distribution curve on the surface of the non-textured valve plate at five different radii; (**c**) The temperature distribution curve on the surface of the square textured valve plate at five different radii.

According to the oil-film-temperature distribution in Figure 8, we choose two different radii values ($r_{1}$ = 0.032 m, $r_{2}$ = 0.033 m) in the inner sealing belt, and three different radii values ($r_{3}$ = 0.039 m, $r_{4}$ = 0.040 m, $r_{5}$ = 0.041 m) in the outer sealing belt. Figure 9a shows the positions of five circles with different radii selected in the sealing belt. Figure 9b shows the temperature distribution curve on the surface of the non-textured valve plate at five different radii. Figure 9c shows the temperature distribution curve on the surface of the square textured valve plate at five different radii. We notice that the highest temperature occurs near a kidney-shaped trough between 200° and 300°, and it is evident that the temperature of the textured valve plate surface is lower than that without texture. By comparing the oil temperature distribution curves of different radii, it can be seen that the overall temperature of the outer sealing belt on the surface of the non-textured surface and the square textured surface is both higher than that of the inner sealing belt. At the same time, the temperature increases with the increase in radii values.

## 4. Friction Characteristics

Compared with the non-textured surface of the valve plate, the texture on the valve plate can improve the load-carrying capacity and friction efficiency of the oil film in different degrees, but the textured structure needs to be adjusted continuously for simulation experiments. Through the comparison of the data results, the best parameters can be found to improve the friction reduction effect as much as possible.

In this section, the friction characteristics values under different valve plate pair parameters and texture structure parameters are calculated, such as the maximum pressure of the oil film, the load-carrying forces, the friction force of the lubricating oil during operation and the friction coefficient.

### 4.1. Influence of Valve Plate Pair Parameters

The initial parameters in Table 1 remain unchanged, except for cylinder block rotation speeds, cylinder block inclination angles, initial film thicknesses and initial oil viscosities.

Cylinder block rotation speeds ω are taken as: 1000 ${r\;\min}^{-1}$; 1500 ${r\;\min}^{-1}$; 2000 ${r\;\min}^{-1}$; 2500 ${r\;\min}^{-1}$; 3000 ${r\;\min}^{-1}$; 3500 ${r\;\min}^{-1}$; 4000 ${r\;\min}^{-1}$; 4500 ${r\;\min}^{-1}$; and 5000 ${r\;\min}^{-1}$.

Cylinder block inclination angles φ are taken as: 0.002°; 0.003°; 0.004°; 0.005°; 0.006°; 0.007°; 0.008°; 0.009°; and 0.010°.

The initial film thicknesses $h_{0}$ are taken as: 17.5 μm; 22.5 μm; 27.5 μm; 32.5 μm; 37.5 μm; 42.5 μm; and 47.5 μm.

The initial oil viscosities $\eta_{0}$ are taken as: 0.0165 Pa s; 0.0265 Pa s; 0.0365 Pa s; 0.0465 Pa s; 0.0565 Pa s; 0.0665 Pa s; and 0.0765 Pa s.

**Figure 10:**
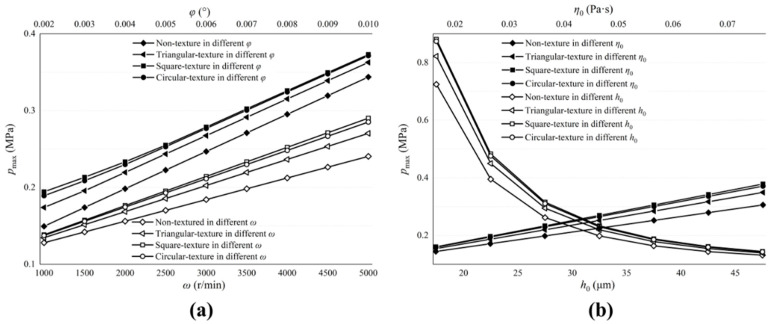
Influence of valve plate pair parameters on maximum pressure of oil film. (**a**) The influence of ω and φ on the maximum film pressure; (**b**) The influence of $h_{0}$ and $\eta_{0}$ on the maximum film pressure.

**Figure 11:**
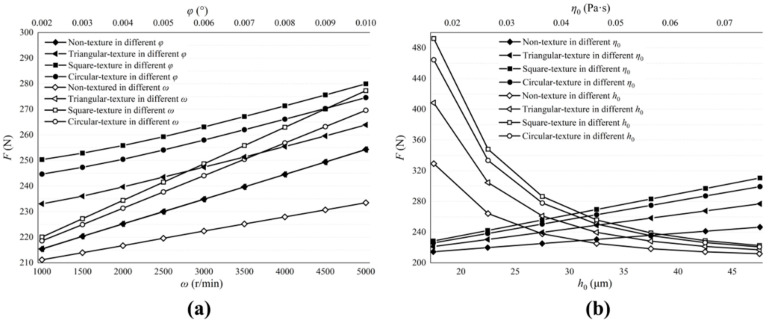
Influence of valve plate pair parameters on load-carrying forces of oil film. (**a**) The influence of ω and φ on the load-carrying forces; (**b**) The influence of $h_{0}$ and $\eta_{0}$ on the load-carrying forces.

Figure 10 and Figure 11 describe the influence of the valve plate pair parameters on the maximum film pressure and load-carrying forces. Figure 10a and Figure 11a show the influence of ω and φ. Figure 10b and Figure 11b show the influence of $h_{0}$ and $\eta_{0}$. Among them, influence factors, ω, φ and $\eta_{0}$ are positively correlated with the maximum oil film pressure and load-carrying forces, while $h_{0}$ is the opposite. The larger the inclination angle of the cylinder block, the stronger the convergence of the oil film thickness in the thin oil film area, which makes the flow hydrodynamic effect more obvious, similar to the increase in cylinder speed, and causes pressure and forces to amplify. 

The biggest impact on the maximum pressure and load-carrying forces are the initial film thicknesses. Reducing thicknesses can greatly increase pressure and forces and, when thicknesses are large enough, the flow hydrodynamic effect is not obvious and almost disappears.

The textured surface of the valve plate can produce larger oil film pressure and load-carrying forces, and the pressure and forces on the circular textured surface is the largest, while the triangular textured surface is the smallest.

**Figure 12:**
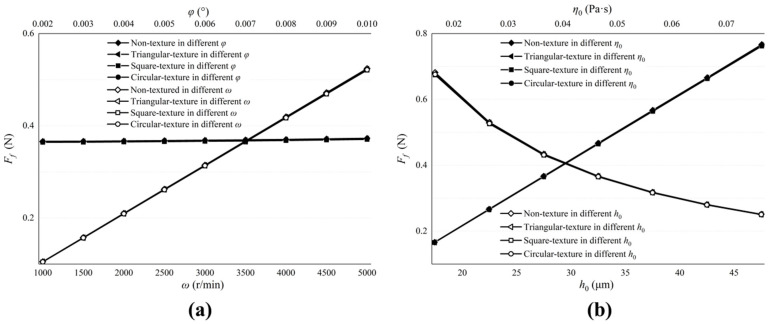
Influence of valve plate pair parameters on friction forces. (**a**) The influence of ω and φ on the friction forces; (**b**) The influence of $h_{0}$ and $\eta_{0}$ on the friction forces.

Figure 12 describes the influence of valve plate pair parameters on the friction forces. Figure 12a shows the influence of ω and φ. Figure 12b shows the influence of $h_{0}$ and $\eta_{0}$. The friction forces increase with the increase in ω and $\eta_{0}$, but when $h_{0}$ increases, the friction decreases. At the same time, we notice that no matter how much φ increases, the change in friction forces is very small. The best way to reduce friction forces is to reduce the initial oil viscosity, but this will lead to smaller load-carrying forces. In actual operating conditions, the appropriate viscosity of the lubricating oil should be selected.

No matter how the parameters of the valve plate pair change, the non-textured and textured surfaces of the valve plate are almost the same for the friction forces.

**Figure 13:**
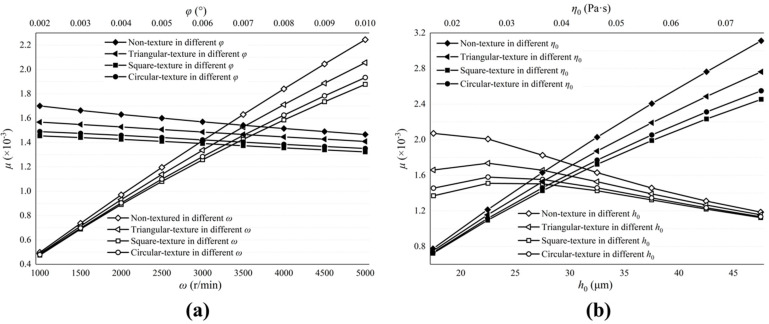
Influence of valve plate pair parameters on friction coefficient. (**a**) The influence of ω and φ on the friction coefficient; (**b**) The influence of $h_{0}$ and $\eta_{0}$ on the friction coefficient.

Figure 13 describes the influence of the valve plate pair parameters on the friction coefficient. Figure 13a shows the influence of ω and φ. Figure 13b shows the influence of $h_{0}$ and $\eta_{0}$. Reducing ω can diminish the friction coefficient and improve the friction efficiency. It has the same effect as reducing the viscosity of the lubricating oil. Although increasing the inclination angle of the cylinder block and the initial oil film thickness can also improve friction efficiency, the result is not obvious and this may lead to an increase in cylinder leakage and a reduction in the volumetric efficiency of the pump.

### 4.2. Influence of Texture Structural Parameters

From Section 4.1, we noticed that the friction characteristics of differently shaped textured valve plate surfaces are distinctive, so it can be inferred that changing the micro-pit parameters will also bring about different friction characteristics. The initial parameters in Table 1 remain unchanged, but the lengths or radii and depths of the micro-pits changed.

**Figure 14:**
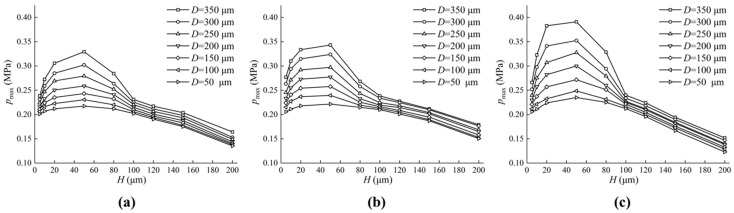
Influence of texture parameters on maximum pressure of oil film. (**a**) The impact of D and H on the maximum pressure of the oil film on the triangular textured surface; (**b**) The impact of D and H on the maximum pressure of the oil film on the square textured surface; (**c**) The impact of D and H on the maximum pressure of the oil film on the circular textured surface.

**Figure 15:**
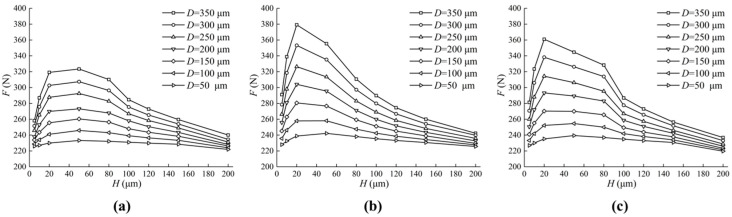
Influence of texture parameters on load-carrying forces of oil film. (**a**) The impact of D and H on the load-carrying forces of the oil film on the triangular textured surface; (**b**) The impact of D and H on the load-carrying forces of the oil film on the square textured surface; (**c**) The impact of D and H on the load-carrying forces of the oil film on the circular textured surface.

Figure 14 shows the effect of different textures on the maximum pressure of the oil film. It can be seen from the figure that, with the increase in texture depth, the maximum pressure of the oil film increases firstly and then decreases. The maximum value of pressure appears near H at 50 μm, and the larger D is, the greater the maximum pressure will be. Compared with Figure 14a–c, it was found that the maximum oil film pressure on the valve plate surface of a circular texture is the largest, followed by that of a square texture. The smallest was that of a triangular texture. The specific texture structure parameters are shown in Table 2:

Figure 15 shows the effect of different textures on the load-carrying forces. The change trend in the load-carrying forces of the oil film are the same as the maximum oil film pressure. When H is between 20 μm and 40 μm, there is a maximum load-carrying force of the oil film, and the larger D is, the greater the forces are. Compared with the triangular textured valve plate surface, the square textured and circular textured valve plate surface provide greater load-carrying forces. 

**Figure 16:**
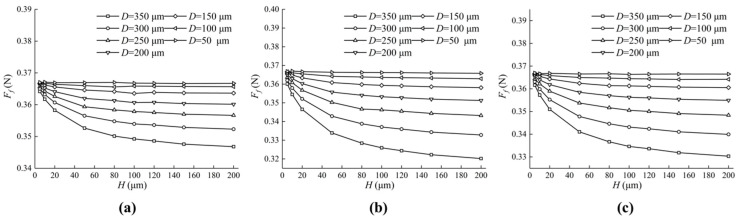
Influence of texture parameters on friction forces. (**a**) The impact of D and H on the friction forces of the oil film on the triangular textured surface; (**b**) The impact of D and H on the friction forces of the oil film on the square textured surface; (**c**) The impact of D and H on the friction forces of the oil film on the circular textured surface.

Figure 16 shows the effect of different textures on the friction forces. Enlarging D and H leads to a decrease in friction forces. However, the friction forces decrease little with the increase in H when D is small enough (50 μm, 100 μm). In addition, the friction forces of the valve plate of a square texture and of a circular texture is smaller than that of a triangular texture. 

**Figure 17:**
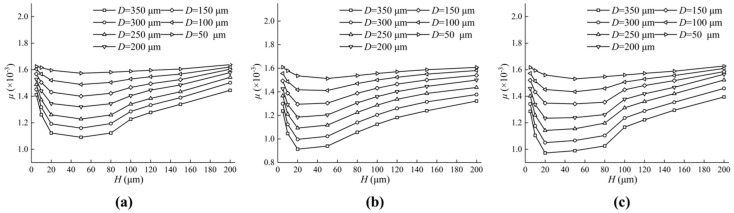
Influence of texture parameters on friction coefficient. (**a**) The impact of D and H on the friction coefficient of the oil film on the triangular textured surface; (**b**) The impact of D and H on the friction coefficient of the oil film on the square textured surface; (**c**) The impact of D and H on the friction coefficient of the oil film on the circular textured surface.

Figure 17 shows the effect of different textures on the friction coefficient. The friction coefficient decreases with the increase in H from 5 μm to 50 μm, and the continuous increase in H brings about the increase in D, which can be seen in Figure 17. When H changes from 20 μm to 40 μm, there is a minimum value of the friction coefficient, which makes the friction efficiency the highest. The larger D is, the smaller the friction coefficient and the higher the friction efficiency. 

Under the same operating conditions, the friction efficiency of the square texture and the circular texture is higher than that of the triangular texture.

## 5. Conclusions

Due to the rotation of cylinder block rotation, the cylinder block inclines to different degrees, which leads to a wedge-shaped oil film with a different thickness. On the inclined side, the oil film thickness reaches the minimum and the hydrodynamic effect is produced under the flow of oil, and the maximum oil film pressure, temperature and elastic deformation are produced in the same region. By comparing the simulation results of the oil film in the textured surface of the valve plate, it is found that the oil film lubrication interface of the valve plate pair with texture can significantly improve the hydrodynamic effect and load-carrying forces. The specific conclusions are as follows:

At the same angular velocity, the larger the radius is, the greater the linear velocity is. Because the friction power is converted into heat energy, the temperature of the oil film increases with the increase in radius. Because the temperature can significantly affect the viscosity of oil and change the pressure distribution and oil film load-carrying forces, the oil film temperature distribution is an important factor affecting lubrication performance.

The maximum film pressure, elastic deformation and temperature can be affected by the initial film thickness, cylinder inclination angle, oil viscosity and rotating speed, and the initial film thickness has the greatest influence on the maximum pressure and elastic deformation, while the inclination angle of the cylinder block has the greatest influence on the maximum oil film temperature.

Under the same operating conditions, the textured surface of the valve plate has larger pressure, elastic deformation, load-carrying forces and a smaller temperature than the non-textured surface.

With the increase in micro-texture depth, the friction efficiency increases first and then decreases, and the friction efficiency reaches the maximum between 20 μm and 80 μm. In addition, the friction efficiency of the textured surface is always higher than the non-textured surface under the same operating conditions.

The friction efficiency can be improved by three different textures. What we noticed is that the friction coefficient of the circular textured surface is smaller, the friction efficiency is higher and the triangular textured surface is the worst.

## Figures and Tables

**Table 1:** Initial parameters.

Parameters	Values
$\omega /\left(r\cdot \min^{-1}\right)$	3500
$h_{0}/\left(\mu m\right)$	32.5
φ/(°)	0.004
$p_{0}/\left(\mathrm{Pa}\right)$	0
$\eta_{0}/\left(\mathrm{Pa}\cdot s\right)$	0.0365
$T_{0}/\left(K\right)$	303
D/(μm)	200
H/(μm)	5
$R_{1}/\left(\mathrm{mm}\right)$	31.8
$R_{2}/\left(\mathrm{mm}\right)$	33.8
$R_{3}/\left(\mathrm{mm}\right)$	37.9
$R_{4}/\left(\mathrm{mm}\right)$	41.9

**Table 2:** Texture structure parameters.

D/(μm)	50	100	150	200	250	300	350	-	-
H/(μm)	5	10	20	50	80	100	120	150	200

## Data Availability

No applicable.
